# A compression transmission device for the evaluation of bonding strength of biocompatible microfluidic and biochip materials and systems

**DOI:** 10.1038/s41598-020-58373-0

**Published:** 2020-01-29

**Authors:** S. R. A. Kratz, B. Bachmann, S. Spitz, G. Höll, C. Eilenberger, H. Goeritz, P. Ertl, M. Rothbauer

**Affiliations:** 1 Institute of Applied Synthetic Chemistry and Institute of Chemical Technologies and Analytics, Faculty of Technical Chemistry, Vienna University of Technology, Getreidemarkt 9/163-164, 1060 Vienna, Austria; 2 Austrian Cluster for Tissue Regeneration, Vienna, Austria; 3 Ludwig Boltzmann Institute for Experimental and Clinical Traumatology, Allgemeine Unfallversicherungsanstalt (AUVA) Research Centre, Donaueschingenstraße 13, 1200 Vienna, Austria; 4 Institute of Solid State Electronics, Faculty of Electrical Engineering and Information Technology, Vienna University of Technology, Gußhausstraße 25-25a, 1040 Vienna, Austria

**Keywords:** Lab-on-a-chip, Biomaterials

## Abstract

Bonding of a variety of inorganic and organic polymers as multi-layered structures is one of the main challenges for biochip production even to date, since the chemical nature of these materials often does not allow easy and straight forward bonding and proper sealing. After selection of an appropriate method to bond the chosen materials to form a complex biochip, function and stability of bonding either requires qualitative burst tests or expensive mechanical multi-test stations, that often do not have the right adaptors to clamp biochip slides without destruction. Therefore, we have developed a simple and inexpensive bonding test based on 3D printed transmission elements that translate compressive forces via manual compression, hand press or hydraulic press compression into shear and tensile force. Mechanical stress simulations showed that design of the bonding geometry and size must be considered for bonding tests since the stress distribution thus bonding strength heavily varies with size but also with geometry. We demonstrate the broad applicability of our 3D printed bonding test system by testing the most frequent bonding strategies in combination with the respective most frequently used biochip material in a force-to-failure study. All evaluated materials are biocompatible and used in cell-based biochip devices. This study is evaluating state-of-the-art bonding approaches used for sealing of microfluidic biochips including adhesive bonding, plasma bonding, solvent bonding as well as bonding mediated by amino-silane monolayers or even functional thiol-ene epoxy biochip materials that obviate intermediate adhesive layers.

## Introduction

**Figure 1:**
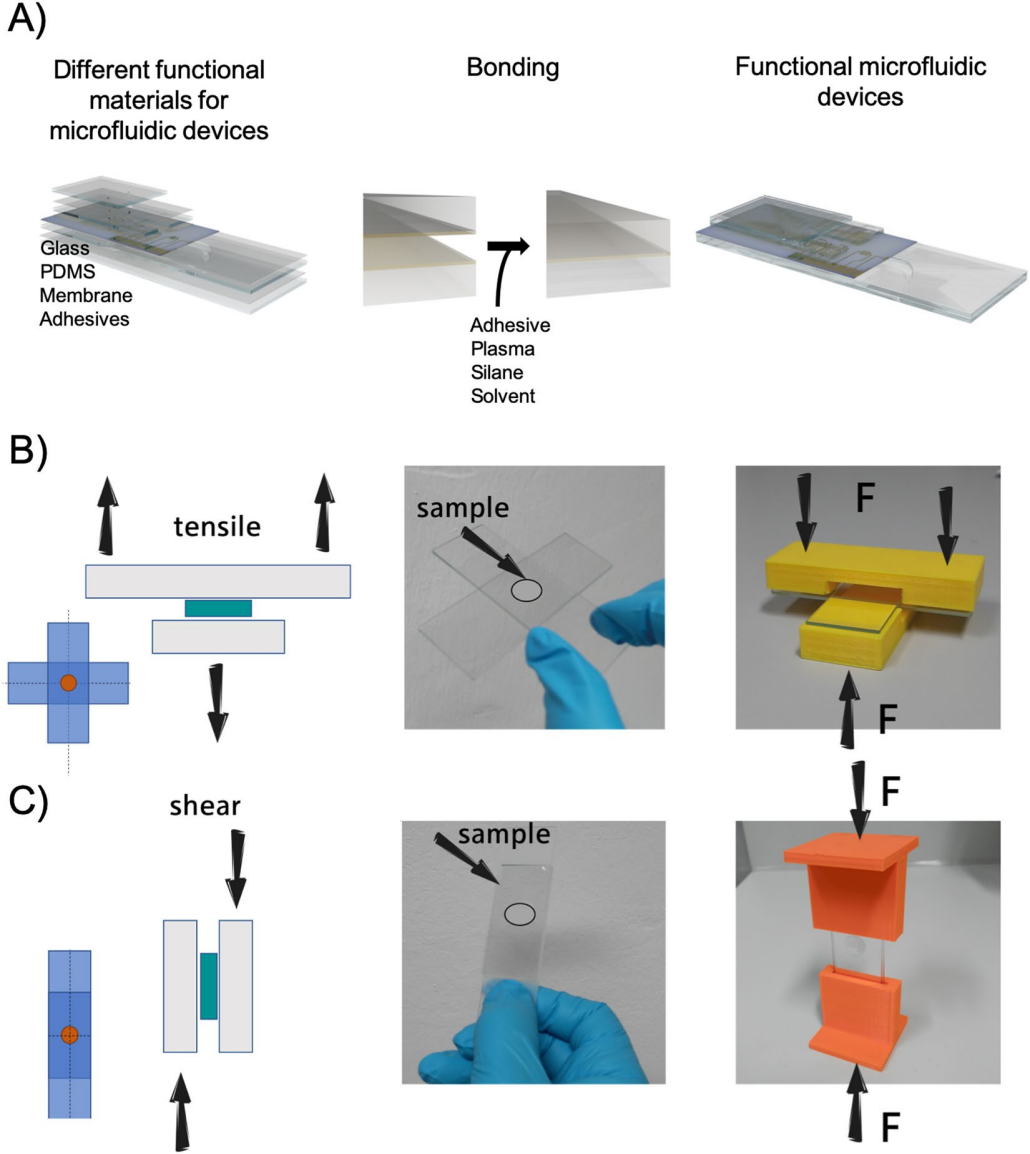
(**A**) Basic assembly steps to fabricate multi-layered multi-material microfluidic chips using state-of-the-art bonding methods. (**B**,**C**) Basic working principle for the 3D printed force transmission devices for (**B**) applied tensile force and (**C**) applied shear force (left schematic; sample turquoise, glass grey), sample with circular bonding area (center image) and sample placed within the force transmission devices (right images).

The emergence of soft lithography, printing techniques and xurography as affordable microfabrication techniques in combination with commercial bench-top size equipment recently has eliminated the dependence on expensive cleanroom facilities for microfluidic and biochip microfabrication^1,2^. Consequently, the interest in cell based microfluidic devices as lab- and organ-on-a-chip technologies has ever since grown for fundamental biological, pharmaceutical and biomedical research using customized and complex chip systems mainly at academic level^3–6^. Around 2000 micromachining techniques were introduced to manufacture and assemble dynamic cell culture devices where one or multiple chambers are interconnected by channels in the microscale to enable a dynamic microfluidic environment^7^. These microfluidic devices aim to simulate the complex microenvironment within the tissue as well as the interaction between multiple types of those through a fluid channel system. Additionally, lab-on-a-chip systems also emerged to enhance the spectrum of point-of-care devices, where the basic aim is maximum functionality (e.g. sample prep, detection, etc.) and portability at minimum costs. For the last two decades the development of more and more complex microdevices that incorporate lab functions such as pumping, valve actuation, mixing and degassing as well as multiple multi-plexed cell culture compartments with integrated biosensors has intensified^5^. Adapting and refining the concept of miniaturized total chemical-analysis systems has brought forth continuously increasing application scenarios where lab-on-a-chip devices can be used for the investigation of cell behavior^8^. Determining factors as the infrastructure, fabrication speed, cost, production accuracy and the material selection restricts the possible fabrication methods as well as material compatibility. At the beginning glass- and silicon-based devices were built to establish microfluidic devices for electrophoresis^9,10^, however replica and injection molding arises in recent years to enable industrial relevant manufacturing. For research purpose fast and inexpensive microdevices production using polydimethylsiloxane (PDMS)^11^, hydrogels^12^, thermoset composites^13^, and thermoplastics^14^ are used. Replica molding is mainly used for fabrication of small series or single prototypes and mainly on academic level. For a more industrial relevant fabrication of microfluidic biochips hot embossing and injection molding polymers as polycarbonate (PC), polymethylmethacrylate (PMMA), cyclic olefin copolymer (COC), polystyrene (PS), polyethylene terephthalate (PETG) and polyvinylchloride (PVC) are used^15^. During fabrication biochip sealing and bonding is a major challenge especially when integrating different material types^16^. Methods can be combined in regards of available chip assembly and bonding procedures as thermal, UV-ozone, plasma-assisted and a few chemical procedures in as well as UV-glues and adhesive tapes^17^. Stable assembly and bonding of microfluidic devices is a key issue for multi-material-composed cell-based biochips (see Fig. 1A). Glues and adhesive bonding may harm cells due to toxicity or do fail to create a stable bond under cell culture conditions^18^. Also, the most frequently applied chemical bonding strategy using plasma and silane chemistry seems not reproducible even though a multitude of different protocols has been established over the years for many silanes^19^. For instance silanization and bonding through 3-Aminopropyltriethoxysilane (APTES) is reported through different protocols in literature which results in bonding strength between 270 kPa and 579 kPa or rather subjective scoring systems such as from 0 to 2 which reflects bonding no bonding at all (0), partially delamination (1) as well as bonded (2)^20–22^.

Reported test systems to evaluate bonding strength are burst pressure test, leakage test, peel-off test and multi-test systems of universal testing machines. To perform material and bonding testing according to international standards, the guidelines of the International Organization for Standardization (ISO), universal testing machines are used to perform normed tensile test of material specimens with defined geometries^23^. Through the defined set-up as sample preparation, fixturing and gauge length this method is not capable to measure the structure of a microfluidic device where often soft polymers are bonded to brittle materials as glass or PMMA etc.^4^. To overcome those restrictions often leakage-, burst pressure- or peel-off test are found in literature to evaluate the physical stability of a microfluidic set up or bonding protocols. Leakage Test are performed by filling up the microchannel with an optical detectable liquid which is pumped with different volume flow through the device. Either the flow or the time is reported when the first leakage appears. Burst tests are performed by applying air pressure to the channel system of the microfluidic device and report the maximum pressure that can be handled by the device. For Peel-off test the samples are mounted in commercially available texture analyzer and the peel-off force is measured^24^. All those methods are applied for each individual microfluidic design for a specific study. Therefore, all the assessed parameters are not comparable due to the vast impact of the individual device geometry. Here, we present an easy and simple device to conduct comparable measurements for tensile and shear bonding stress for microfluidic Devices (see Fig. S1). To our knowledge this is the first study where different material set-ups, which are most commonly used for cell-based microfluidics based on set-ups with biocompatible materials, are compared on bonding specific level and geometry independent. This study compares the bonding strength of relevant biocompatible materials including regards of adhesive, plasma treated, silane and solvent bonded material combinations of interests.

## Results

### Physical characterization of the 3D printed tensile/shear force transmission system

**Figure 2:**
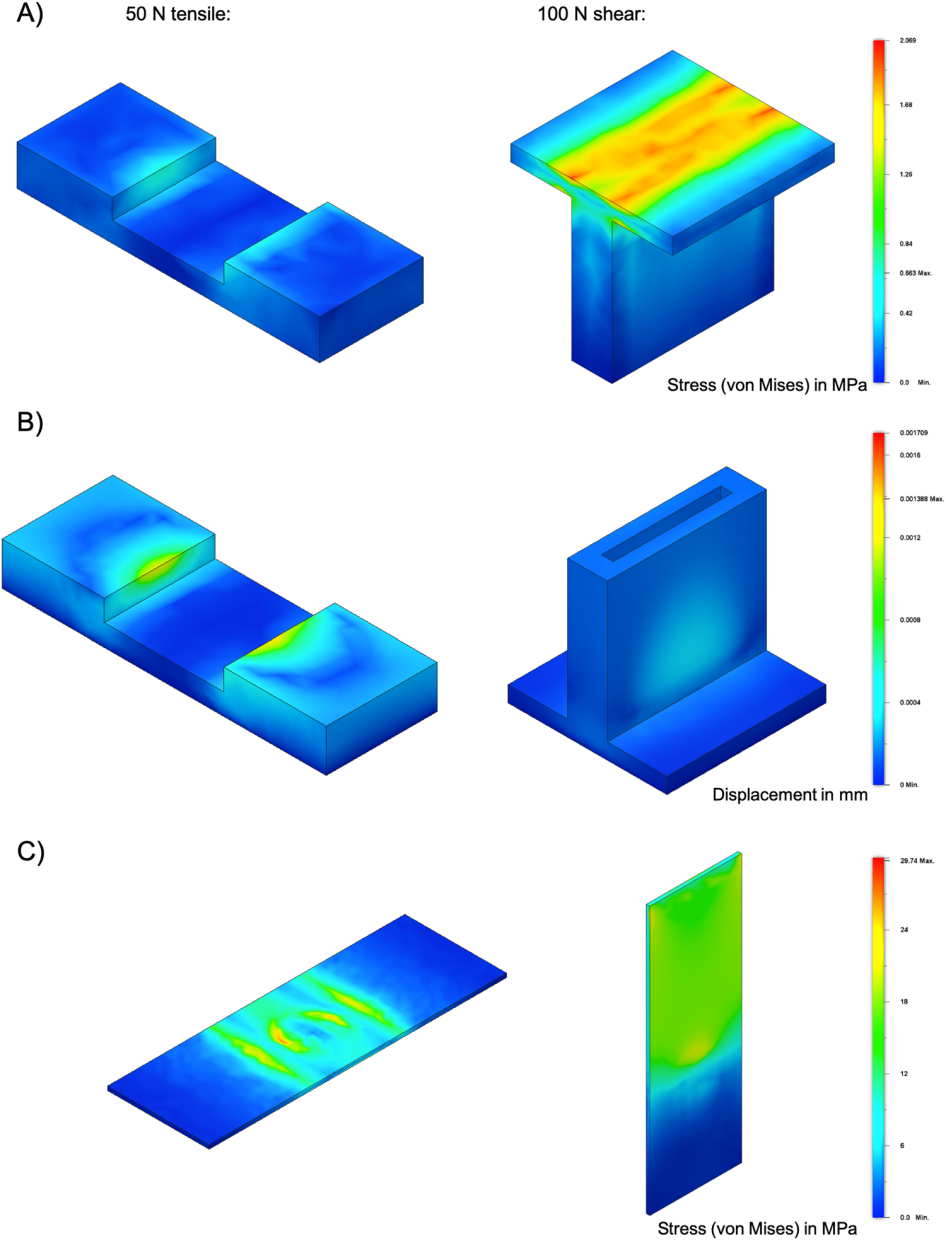
Simulation of (**A**) stress (von Mises) and (**B**) deformation in mm for the tensile measuring device (left side) with 50 N applied and the shear measuring device (right side) with 100 N applied on the upper part of the transmission devices. (**C**) Stress (von Mises) on the sample carrier (glass) for both shear and tensile stress transmission devices at a simulated circular bonding area of 0.5 cm^2^ placed at the center of the slide.

To evaluate the degree of three-dimensional stress impact of applied load that will be exerted on the test substrates during the tensile and shear force bonding evaluation, the von Mises stress within the device as well as the deformation was simulated *in silico*. Results as shown in Fig. 2A indicate that the maximum stress in the tensile device is 0.66 MPa and 2.07 MPa for the shear stress device, respectively. These forces result in a maximum displacement of 1.4 µm for the tensile device and 1.7 µm for the shear device as shown in Fig. 2B. Both the maximum stress and displacement occur at the inner edges of the area of the support of the tensile transmission device which is directly in contact with the sample substrate surface. For the shear device the maximum stress and displacement takes place in the middle part of the surface where force is applied. In turn, the maximum stress for the sample carrier (object slides) is 29.7 MPa at the edge of the circular bonding sample (tensile) respectively 21.6 MPa (shear) (see Fig. 2C).

The simulation, as shown in Fig. 2, the 3D printed tensile/shear force transmission system experiences during load minimal deformation in the range of 2 µm, during load which has no influence on the measuring of the bonding strength of the sample of interest. Furthermore, the weight of measuring device on top of the sample carrier (16 g for tensile device, 19 g for shear device) has a neglectable impact on the bonding compared to the magnitude of the bonding strength with in MPa. Therefore, the transmission devices directly transmit the force to the bonding interface between two substrates where its manifests in either tensile or shear stress as a result of mechanical compression (see Fig. S2). What has to be noted is that most likely in the regions of maximum stress as discussed above, brittle materials such as glass will break when the bonding strength exceeds the mechanical strength of the substrate material. Furthermore, the dimensions of conventional glass slides, which are largely used for cell-based biochips due to the compatibility with conventional microscopes, cell adhesion and biocompatibility, give several design restrictions to the device. With the given design conventional microscope slides can be mounted and supported to prevent breaking of the glass but also enable a defined delamination area to be investigated.

### Influence of sample geometry of tensile and shear force transmission

**Figure 3:**
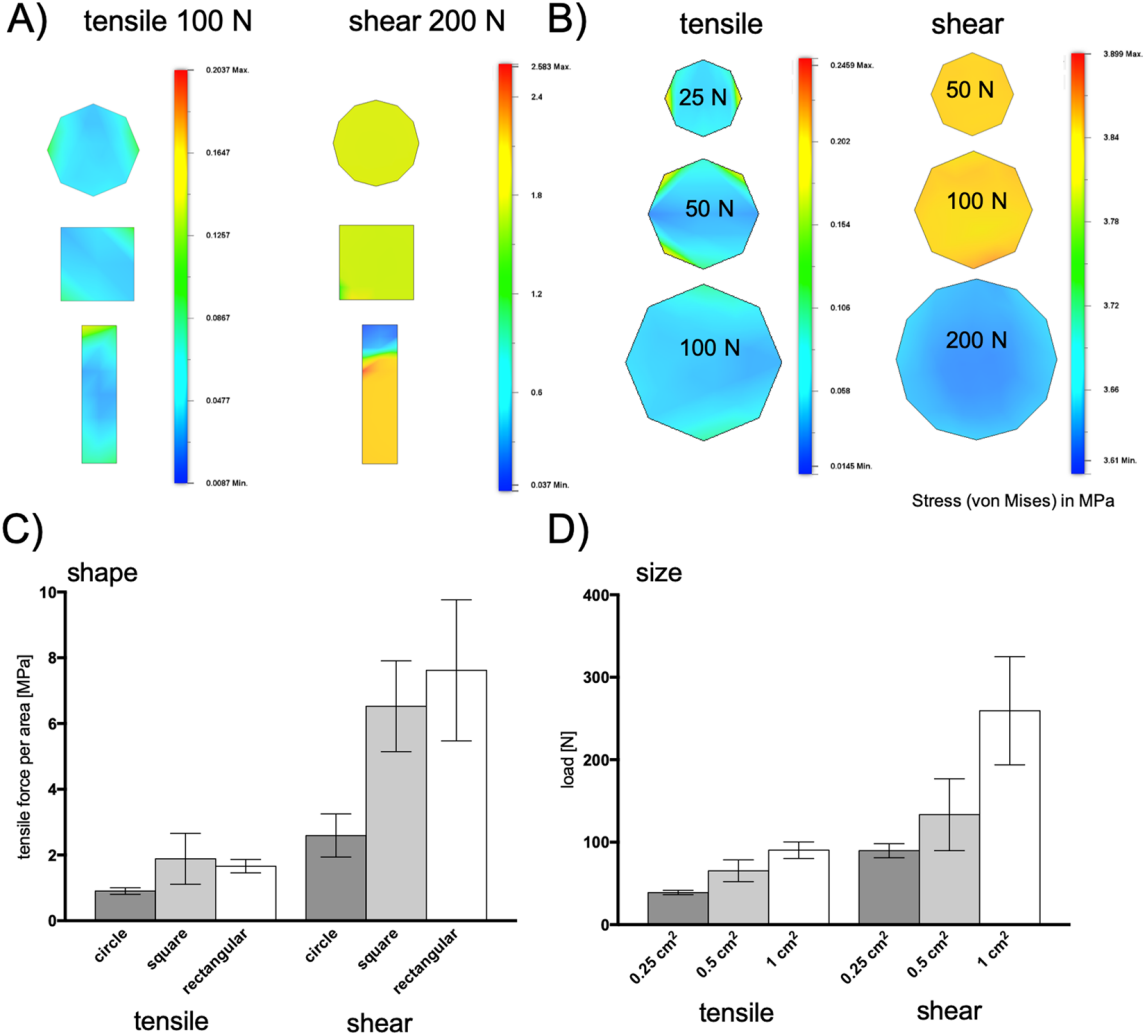
Simulations and experimental data on the influence of (**A**,**C**) sample shape of 1 cm^2^ bonding area as well as (**B**,**D**) bonding area on tensile stress and shear stress distribution. Pressure sensitive double sided adhesive ARcare 90445 was tested as bonding mediator to glass. Data points are presented as mean values ± SD for n = 3.

First the influence of sample shape and/or size was simulated to identify optimal sample geometry for evaluating the bonding strength of different materials and combinations of hybrid materials frequently found for microfluidic device fabrication. A comparison between different shapes as circular, rectangular and square with the same area (see Fig. 3A) shows that circular shaped samples have the most homogenous stress distribution for both tensile and shear stress. In contrast, the rectangular shaped sample (5 × 20 mm) indicates the highest stress in both cases with 1.72 MPa (tensile) and 2.58 MPa (shear) as well as an inhomogeneous stress distribution. For the square-shaped sample the maximum stress is 0.2 MPa and 2.28 MPa. For the circular sample the maximum stress is 0.138 MPa and 1.83 MPa. In contrast the minimum stress that occurs during both applications (tensile and shear) are observable for the circular sample 0.02 MPa and 1.8 MPa, for the square sample 0.09 MPa and 0.94 MPa and for the rectangular sample 0.001 MPa and 0.037 MPa. Beside the overall sample geometry, next the influence of increasing bonding area was simulated and measured for circular shapes with a base area of 0.25 cm^2^, 0.5 cm^2^ and 1 cm^2^. Figure 3B shows that with increasing size stress gets distributed uniformly within the sample. For 0.25 cm^2^ sample size, stress is min. 0.04 MPa and max. 0.25 MPa tensile as well as min. 3.84 MPa tensile and max. 3.87 MPa shear. For the 0.5 cm^2^ sample the minimum stress is 0.015 MPa and the maximum stress is 0.23 MPa for tensile load and min. 3.83 MPa and 3.9 MPa for the impact of shear force. Samples in the size of 1 cm^2^ exposed to 100 N of tensile force show a stress between 0.013 MPa and 0.13 MPa as well as 3.61 MPa up to 3.65 MPa for a shear load of 200 N. To validate the results of the simulations, next bonding strength of similar sample with the same geometry of ARcare 90445 biomedical double-sided adhesive tape bonded between two glass slides was evaluated with the 3D printed force transmission devices. As shown in Fig. 3C the bonding strength of different geometries are 0.90 MPa for circular shaped samples, 1.89 MPa for square samples and 1.66 MPa for rectangular shaped samples in regards for tensile stress. For shear stress the circular sample fails at 2.59 MPa, the square one at 6.5 MPa and the rectangular sample at 7.62 MPa. Figure 3D shows the influence of the different bonding area sizes on bonding strength of samples from ARcare 90445. For a sample with a bonding area of 0.25 cm^2^, the sample withstands an impact of 39 N when a tensile load is applied and an impact of 90 N when shear force is applied. The 0.5 cm^2^ sized sample fails at a tensile force of 65 N and at 133 N under shear force impact. The sample of the size of 1 cm^2^ shows a failure at 90 N for tensile impact and 259 N for a shear impact. Overall, geometry has a big impact on the maximum load a bonding can carry. Especially when shear tests are performed the area along the direction of the shear impact has a big influence on the magnitude of the force to failure, (compare Fig. 2C with p < 0.05 for shear impact on the circular shape) because less edge is directly exposed to the impact front. The impact of the edge of the sample can be also found when the sample shape is kept constant, but the dimensions are changed (see Fig. 2B). For tensile stress tests a more homogenous stress distribution can be found with the increase of the diameter of the circular shaped sample. The bigger the samples get the smaller the ratio between the length of the edge and the surface becomes. This effect can also be seen in Fig. 2D where the strength doesn’t increase proportionally with the surface of the bonding most notably for the tensile impact. For the assessment of the bonding strength in this study 0.5 cm^2^ was chosen as sample size to assure that the overall bonding strength is smaller than the maximal load glass slides can carry mounted in the device (84 N for tensile load and min. 1000 N for shear load). The simulations show that size and shape have a big impact on the investigation of specific biochip bonding. Therefore, it is necessary to establish a comparable value for the bonding of different combinations of biochip materials, independent from a particular microfluidic design.

### Assessment of bonding strategies for biocompatible microfluidic and biochip materials using the 3d printed compressive 3D devices

In a final experimental study, most commonly applied bonding strategies were tested for shear and tensile force-to-failure to biochip substrates including glass, cyclic olefins, polymeric track-etched membranes as well as advanced functional biochip material OSTEmer (thiol-ene epoxy polymer) including pressure sensitive adhesives (acrylic ARcare and silane-based ARseal), oxygen plasma bonding (PDMS-PDMS and PDMS-glass), solvent bonding (COC-COC and COC-COP), as well as silane-assisted chemical bonding (amino-silane with PDMS, PET, PC). The bonding was assessed directly after the procedure as well as after 7 days under standard cell culture conditions of 37 °C and 100% humidified atmosphere, which represents a rather harsh environment for material bonds.

**Figure 4:**
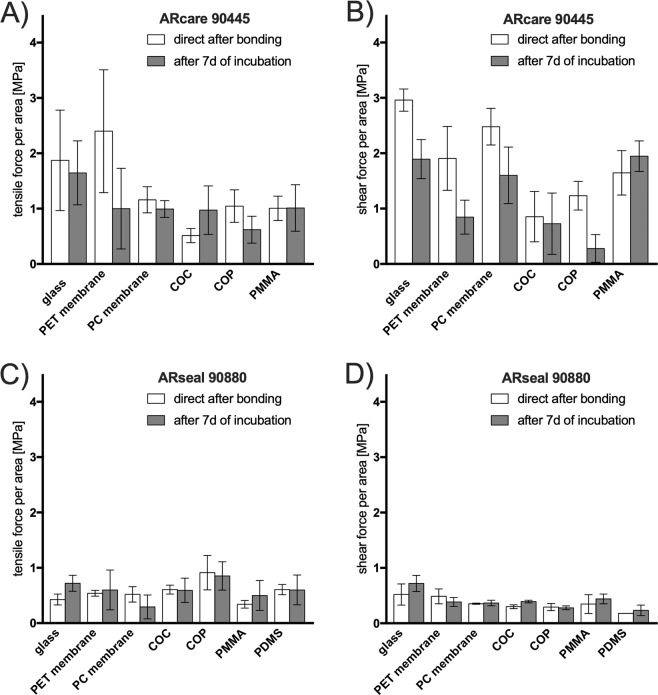
Tensile stress and shear stress (force to failure) for biochip bonding mediated by an intermediate layer of pressure sensitive adhesives ARcare 90445 (**A**,**B**) and ARseal 90880 (**C**,**D**) to a variety of biochip materials Data points are presented as mean values ± SD for n = 3. Samples were subjected for 7 days to 100% humidity and 37 °C simulating cell culture conditions.

Firstly, commercially available biocompatible pressure sensitive adhesive tapes offer an easy and fast way to bond different functional material such as flexible membranes, glass, silicone and polymers to assemble multi-material/hybrid devices to build up multiplicity set-ups^25^. Results in Fig. 4A–D show obtained tensile force and shear force-to-failure directly after bonding (applied pressure of 2 kN/cm^2^) and after 7 days under cell culture incubation conditions (37 °C and 100% humidity). 0.5 cm^2^ sheets of pressure sensitive adhesive tapes were layered between two glass slides containing various integrated materials such as PET and PC membranes, COC, COP and PMMA as well as PDMS for ARseal 90880. The values shown in Fig. 4A–C reveal that ARcare 90445 can handle a higher force impact for any material combination in regards of tensile and shear load, while the ARseal 90880 adhesives showed a weaker bonding strength towards the different materials. In contrast ARseal 90880, with a silicone based adhesive, bond to PDMS with up to 0.60 MPa (tensile) and 0.23 MPa (shear). Between directly after bonding and after 7-days of incubation ARcare 90445 showed a decrease of the bonding strength to all materials except COC (tensile) and PMMA (shear). ARseal 90880 shows an increase of the bonding strength of all materials except for PC (tensile) and PET (shear). Additionally, 6-fold higher shear forces were necessary to delaminate ARcare 90445 when compared to ARseal 90880 (see Fig. 4D) from glass, a widespread used material in microfluidics. PDMS even more often used either through soft lithography or xurography, can be bonded to different materials via ARseal 90880 which overcomes the drawback that PDMS does not bond to different polymers without any further chemical treatment. The investigated pressure sensitive adhesives show a tensile bonding strength between 0.5 MPa (ARseal 90880) up to 1 MPa (ARcare 90445) for six different materials used frequently for microfluidic devices. Notably ARseal 90880 sticks to PDMS and is thereby a way to overcome the widely reported bonding problem of bonding PDMS to different materials besides glass, silicon and PDMS. However, due to the more viscous nature of the incorporated thicker adhesive layers for ARseal compared to ARcare the bonding shows flexible deformation which is sensitive especially to the impact of shear (see Fig. 4D).

**Figure 5:**
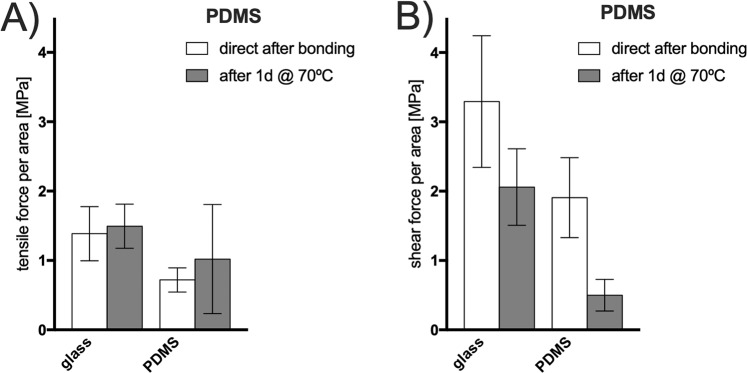
(**A**) Tensile stress and (**B**) shear stress (force to failure) for air plasma bonding of PDMS to PDMS and PDMS to glass for (**A**) tensile stress and (**B**) shear stress using the compressive transmission devices. Data points are presented as mean values ± SD for n = 3.

Secondly, oxygen plasma-assisted bonding of PDMS biochips is the golden standard in cell-based microfluidic devices as well as droplet generators due to the easy and straight-forward fabrication through soft lithography^2^. Mostly the micro-structured PDMS layer is either bonded through air or oxygen plasma activation to another PDMS or glass substrate to seal the device. To assess the capacity of plasma bonding between PDMS and glass circular samples of 0.5 cm^2^ base area are bonded between two glass slides. For the assessment of PDMS to PDMS bonding first two circular samples (0.5 cm^2^) are bonded on two glass sides and afterwards the two PDMS surface are bonded to each other to complete the multi-layered assembly. As Fig. 5 shows the bonding strength between glass and PDMS is about 1.5–2 times higher compared to PDMS bonded to PDMS (p < 0.1 direct after bonding). Glass to PDMS shows a bonding strength of 1.38 MPa directly after bonding for tensile impact where PDMS to PDMS withstands only 0.72 MPa. After prolonged thermal curing of the samples for another 24 h at 70 °C the bonding strength increased for tensile stress but decreased for shear stress for PDMS-PDMS bonding. The results show that PDMS bonds stronger to glass through plasma activation compared to PDMS surfaces. PDMS shows similar bonding strength as ARcare 90445 presented in the former section. Through the curing of PDMS at 70 °C for 24 h the activated surface can establish even more covalent bonds compared to a fresh bonded biochip assemblies directly after plasma activation. The weaker PDMS-PDMS bonding compared to PDMS bonded to glass can be explained by the air oxygen plasma setup that shows weaker bonding results compared to adjustable oxygen plasma chambers that can increase the oxygen content within the vacuum chamber above the atmospheric oxygen content, therefore resulting in formation of more covalent surface groups available for bonding^26^.

**Figure 6:**
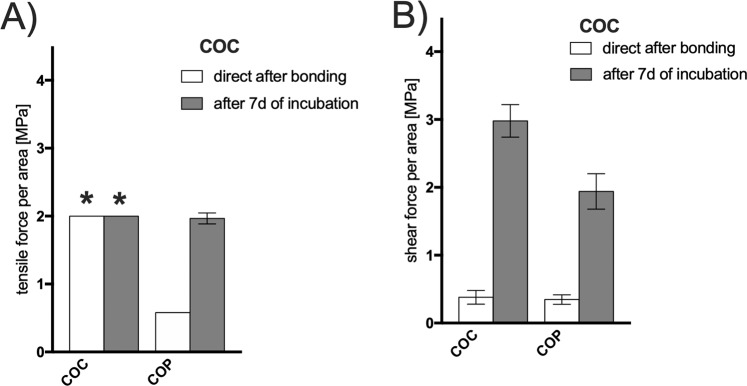
(**A**) Tensile stress and (**B**) shear stress (force to failure) for solvent bonding of COC to COC and COP. *Indicates that value above 2 MPa was not measurable because of the deformation (bending) of the COC sample carrier. Data points are presented as mean values ± SD for n = 3. Samples were subjected for 7 days to 100% humidity and 37 °C simulating cell culture conditions.

Thirdly, most microfluidic devices built of advanced industrial polymers can be easily bonded via solvent-assisted surface activation, where a solvent softens the polymer surface and through the applied pressure a polymer chain interdiffusion occurs at the bonding junction while the solvent evaporates. This is the method of choice because this process does not introduce foreign chemical species (e.g. adhesive layers and reactive glues) and the solvent is completely evaporated during drying of the biochip assemblies^27^. Two particular polymers of interest are cyclic olefins including Topas®(COC) and Zeonor ®(COP) because of high optical transparency, good solvent stability and high mechanical stability. Both materials can be bonded via Cyclohexane diluted Acetone fumes that dissolve the surface layers and create the material bond by compression and thermal drying. Figure 6 shows the bonding strength of COC to COC and to COP after solvent bonding of a COC foil to COC and COP polymeric object slides (76 × 26 × 1 mm). In the chosen setup it was not possible to test the force-to-failure of COC to COC due to the fact that the sample carrier bended heavily before the solvent bonding delaminated indicating a very strong and stable bond for both incubation conditions. All other bonding strengths increased over the 7-day incubation up to 8-fold (shear impact for COC bonding, with p < 0.01 for all measured values). Through the incubation time residual amounts of the solvent can evaporated and enhanced the establishment of additional bonds. Microfluidic devices based on COC and COP bonded based on solvents are as demonstrated highly applicable for a harsh cell culture environment because the bonding is resistant to the high humidity and the increased temperature.

**Figure 7:**
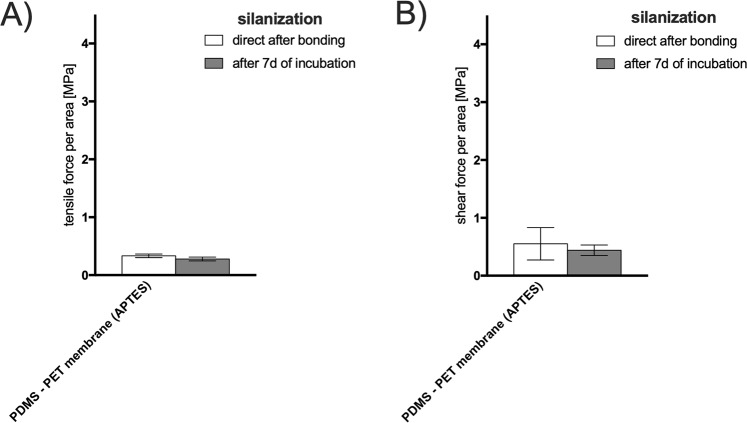
(**A**) Tensile stress and (**B**) shear stress (force to failure) for amino-silane-mediated bonding plasma-assisted of PDMS to porous PET membranes. Data is expressed as mean values ± SD for n = 3. Samples were subjected for 7 days to 100% humidity and 37 °C simulating cell culture conditions.

Using silane chemistry, PDMS biochips can be bonded together with porous membranes (PC or PET) through chemical pretreatment of the plasma-activated membrane surfaces with organofunctional silane molecules^21^. Hydroxyl groups which interact and displace the alkoxy groups on the silane from a covalent -Si-O-Si- bond between the different functional materials thus providing a bond of incompatible material types. For the next experimental setup, PDMS sheets in the size of 26 × 26 mm were bonded to glass as described before. Afterwards a PET membrane was bonded between the PDMS sheets by amino-silanization process. Figure 7 shows that silanization enables bonding between PDMS and PET with a bonding strength up to 0.27 MPa (tensile) and 0.44 MPa after 7d of incubation. Through the incubation period the tensile and shear strength decreased by 18% and 20%, respectively but showed no significance in change. Even though frequently used, amino-silanization yields low bonding strength that is sufficient enough to withstand harsh cell culture conditions over 7 days for standard applications at low fluid pressure.

**Figure 8:**
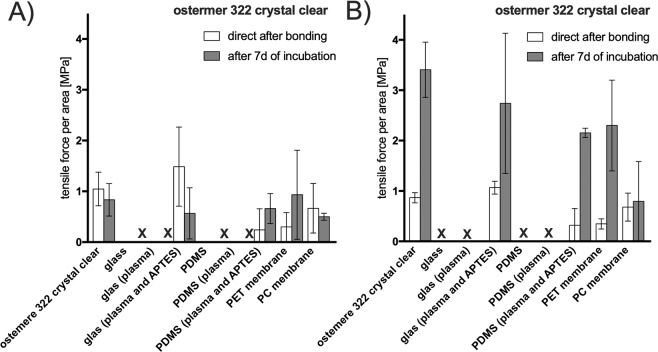
(**A**) Tensile stress and (**B**) shear stress (force to failure) for bonding of OSTEMER 322 crystal clear to various biochip materials. “X” indicates delamination of the bond. Data points are expressed as mean values ± SD for n = 3. Samples were subjected for 7 days to 100% humidity and 37 °C simulating cell culture conditions.

As last type of biochip bonding strategy, a functional and advanced thiol-ene biochip polymer containing self-bonding epoxy groups. Ostemer 322 crystal clear is a two-component thiol-ene-epoxy for reactive two-step injection molding for straight forward integration of other materials. As Fig. 8 shows, Ostemer 322 can be bonded easily to other Ostemer layers as well as PET and PC membranes during the curing process. In contrast, Ostemer weakly bonds to pristine glass and PDMS. To improve bonding to these two materials, a combination of plasma activation and amino-silanziation process, as described before, results in high tensile strength for the silane-based bonding to glass with 1.48 MPa direct after bonding. An incubation period of 7d at 37 °C and a humidity of 100% did not change the tensile strength for any material combination significantly (p > 0.2). In contrast, shear strength increased over the 7d of incubation with a factor of up to 4 times for Ostemer 322 crystal clear bonded on Ostemer 322 crystal clear as well as for amino-silanized PDMS and PET (p < 0.05). Even though cell culture conditions over 7 days influenced the bonding strength, no delamination of the established bonding interface was observable in any material combination. Overall, functional biochip materials with surface groups for improved bonding can make fabrication of multi-layered biochips comprising of different organic and inorganic polymers easier. However, Ostemer 322 clear displays scavenging of dissolved oxygen with in microfluidic devices^28^, which should be considered as unwanted effect for cell-based biochip applications.

### Assessment of bonding strength for advanced cell-based microfluidic, biochip systems and interfaces

**Figure 9:**
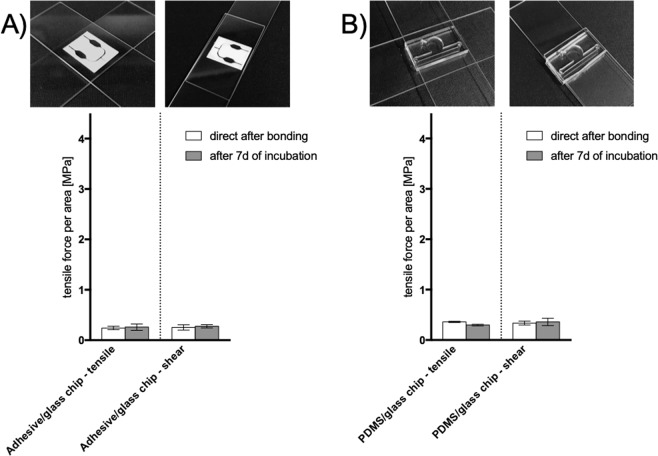
(**A**) Tensile stress and shear stress (force to failure) for bonding for (**A**) glass and biocompatible pressure sensitive based chip (**B**) glass/PDMS based chip. Data points are expressed as mean values ± SD for n = 3. Samples were subjected for 7 days to 100% humidity and 37 °C simulating cell culture conditions.

After evaluating the bonding strength between different combinations of biocompatible materials, entire advanced cell-based microfluidic and biochip systems are tested. To investigate non-invasive real-time oxygen biosensing in two- and three-dimensional microfluidic cell models a bio chip composed out of glass and biocompatible pressure sensitive adhesive tapes was developed^29^. The single microfluidic system within the dimensions of 20 × 15 mm was rebuilt for the bonding testing device, mounted and tested (see Fig. 9A). The system composed out of ARseal 90880 and ARcare 8259 bonded between glass slides showed an average tensile bonding strength of 61 N with results in a bonding strength of 0.24 MPa directly after the bonding process and 65.3 N (0.26 MPa) for the given system with a bonding surface of 2.52 cm^2^. The shear bonding strength reached a magnitude of 63.3 N (0.25 MPa) directly after bonding and 69.7 N (0.28 MPa) after 7 days of incubation. The delamination occurred between the adhesive layer of ARcare 90880 and the non-adhesive surface of ARcare 8259 for the tensile delamination. Through the incubation environment the bonding strength was only increased by 7% for tensile bonding strength and 9% for shear bonding strength. As showed above in Fig. 4C,D ARseal 90880 shows similar behavior for tensile and shear stress due to the more viscous nature of the incorporated thicker silicone adhesive layers compared to the acrylic medical grade adhesive attached to the glass surface. These results show that the crucial interface is the bonding between the two tapes with a lower magnitude compared to bonding between ARseal 90880 and glass. Moreover, during shear stress the delamination was observed between ARcare 90880.

A PDMS glass biochip for engineering three-dimensional pre-vascular networks within fibrin hydrogel for controlling reciprocal cell signaling was developed by Bachmann *et al*.^30^. A single microfluidic system, out of PDMS was bonded through plasma activation between two glass slides (see Fig. 9B). The PDMS glass biochip showed an average tensile bonding strength of 76.6 N with results in a bonding strength of 0.33 MPa for the given system with a bonding surface of 2.13 cm^2^. After 7 days under cell culture conditions the microfluidic system can handle a tensile stress of 63.3 N (0.29 MPa). The shear bonding strength reached a magnitude of 71.6 N (0.33 MPa) directly after bonding and 76.6 N (0.36 MPa) after 7 days of incubation. Incubation caused the bonding strength to decrease by 17% for tensile bonding stress and increased by 7% for shear bonding stress.

Compared to the magnitude of direct boned PDMS foil as shown in Fig. 5 the replica molded device showed a lower force-to-failure. The difference in the bonding strength is caused by differences in the material composition (ratio of current agent and silicon base).

Depending on the features of the microfluidic device different materials has to be combined to establish a defined microenvironment. Those materials define the bonding procedure which can result in either covalent or non-covalent bonding of the different substrates. Adhesive bonding is suitable for material combinations with different thermal expansion coefficients for example^31^. Plasma bonding for example is the mostly used to combine glass with PDMS to form covalent bonding^32^. If plasma activation is not possible because of material (PET or PC) also silanization can be used to establish covalent bonding for PDMS^21^ or to bond Thiol–ene–epoxy thermosets to glass^33^. Through the soft nature of PDMS and the stable use in cell culture conditions (37 °C and 100% humidity) the linear dependence of adhesion strength on temperature for soft membranes has to be taken into account. Through the temperature caused ripples on the interface the process can be remodeled understand the temperature sensitive kinetics of soft membrane adhesion. Furthermore, surface roughness as well as local composition and pre-existing defects has to be taken into account^34^. For the solving bonding of COC advantage is taken of the polymers solubility in Cyclohexane to reach entanglement of polymer chains on the bonding surface. Through the solvation, the polymer chains become mobile and diffuse across the solvent phase. This process leads to an extensive intertwining of the molecules between the polymers leading to sufficient strong bonding^35^.

The delamination of such sealings can be described as a stochastically traction-separation^36^. Here bonding on atomistic and molecular level are used to describe the meso-scale traction-separation as a response of quasi-static loading. The model leads to a varying of bonding probability as a function of separation for tensile stress.

## Discussion

Bonding of biochips layers to create a stable seal is a major challenge in the successful development of functional multi-layered biochips, cell-based microfluidics, lab-on-a-chip systems as well as more recently organ- and body-on-a-chip systems. Overcoming bonding issues concerns the whole microfluidic community and is an arising issue from the beginning of the microfluidic field up to now^32^. Frequently materials used for chip fabrication are made from organic as well as inorganic materials and the combination thereof is restricted by incompatibility of the chemical nature of the materials in combination with the bonding approach. Material combinations have to be chosen accordingly to the intended application of the microfluidic system as biocompatibility, tolerance, gas permeability or transparency. Those design constraints allow only certain manufacturing methods to be carried out. Furthermore, advanced cell-based microfluidic systems and biochip often are composed of several material with in one device to enable a specific microenvironment and necessary features as glass for transparency or silicon for biocompatibility^37^. Beside silicon and glass, thermoplastics offer board possibilities of substrate bonding options which also arise a set of unique challenges for proper sealing processes^35,38,39^. For that reason, it is necessary to evaluate and compare bonding and sealing strength of multi material composed microfluidic devices and biochips.

Therefore, easy and straight forward evaluation of bonding can improve overall yield of the fabricated biochips and can also facilitate comparability of bonding quality with data sets reported in literature. To obviate the need for either qualitative burst tests frequently applied in the microfluidics community or peel-off tests performed with expansive mechanical multi-test systems, we have developed 3D printed transmission devices for shear and tensile force-to-failure tests on biochip materials in standardized object slide format. The simple yet innovative character of these transmission elements lie in the fact that any compressive force applied by manually or via a hydraulic or hand press is directly transformed into shear or tensile forces. In the current study, a compression load cell with digital display beneath the transmission devices was used for data acquisition. Overall, we could demonstrate the influence of bonding area and geometric shape is an important factor to be considered for establish a comparable methodology to assess the tensile and shear bonding strength of biochip materials in a standardized and most importantly comparable way. In a broad screening effort, we compared state-of-the art biochip materials and bonding strategies right after bonding as well as after exposure to a harsh humid cell culture environment to evaluate stability of each individual bonding approach. As proof-of-principle for the proposed compressive bonding evaluation method several state-of-the-art bonding approaches used for sealing of microfluidic biochips including adhesive bonding, plasma bonding, solvent bonding as well as bonding mediated by amino-silane monolayers or functional thiol-ene epoxy biochip materials as well as entire cell-based biocompatible microfluidic and biochip systems

## Material and Methods

### Device design and manufacturing

The devices to assess the different bonding strength in regards of tensile and shear stress was designed with Fusion 360 (Autodesk) as a CAD model. The model was converted with PrusaSilcer (Prusa Research s.r.o.) and printed from PLA with an Original Prusa i3 MK2S printer (Prusa Research s.r.o.). The following printer settings were used: layer height of 0.2 mm, perimeters of 3, top and bottom solid layer of 2 and a rectilinear fill pattern with 20% density. The printing speed was: perimeters 60 mm/s, infill 80 mm/s and travel 130 mm/s. The PLA filament has a diameter of 1.75 mm and is extruded with 220 °C and printed on the bed with 55 °C. One part of the tensile device has a mass of 16 g and one part of the shear device has 19 g.

### Simulations

The modelling and simulation of the stress within the CAD model of the device and the samples was conducted with Fusion 360 (Autodesk) and the build in Static stress simulation tool. The glass slides were modeled with the dimensions of 76 × 26 × 1 mm and material glass, the samples were modeled as rubber/silicone with thickness of 0.05 mm (circular sample with a radius of 2.82 mm (≙0.25 cm^2^), 3.99 mm (≙0.5 cm^2^), 5.64 mm (≙1 cm^2^), rectangular sample 5 × 20 mm, square shaped sample 10 × 10 mm). The device was modeled as PC/ABS plastic. The interfaces of the sample to the glass slides was modeled with in the software as bonded to simulate the highest possible impact of the applied force on the device with the aim to understand the deformation of the device and not the delamination process. To simulate the tensile stress a load of 100 N for 1 cm^2^ samples, 50 N for 0.5 cm^2^ and 25 N for 0.25 cm^2^ samples was applied and 200 N for 1 cm^2^ samples, 100 N for 0.5 cm^2^ and 50 N for 0.25 cm^2^ for simulating shear stress.

### Sample preparation

Glass slides from VWR, Austria were washed by ultra-sonic with Hellmanex III (Sigma Aldrich Z805939) 2% solution for 5 min at room temperature afterwards flushed with deionized water, than washed by ultra sound with isopropanol for 5 min at room temperature, flushed again with deionized water and finally washed by ultra sound with deionized water for 5 min at room temperature. To assess the maximum load the glass slides, the glass slides has been placed with in the device without any bonding sample in between. The glass slides can carry a load of 84 N (n = 5) in the tensile device and do not break in the shear device for forces up to 1000 N.

For pressure sensitive adhesives ARcare 90445^®^ (Adhesive Research, UK), a clear pressure sensitive double-sided adhesive tape out of a 25.4 μm thick polyester film with AS-110 acrylic medical grade adhesive (27.94 μm on each side) with results in a total thickness of 81.28 μm (with liners 182.88 μm) as well as ARseal 90880^®^ (Adhesive Research, UK) is a clear pressure sensitive double-sided adhesive tape out of a 50.8 μm thick polypropylene film with SR-26 silicone adhesive (45.72 μm on each side) with results in a total thickness of 142.24 μm (with liners 243.84 μm) was used.

All shapes in the pressure sensitive double-sided adhesive tapes and Polydimethylsiloxane (PDMS) foil (Super clear 0.5 mm MVQ Silicones GmbH) were designed with CutStudio (Roland). Plotting was performed by a CAMM-1 Servo GX-25 (Roland) with a ZEC-U5032 (Roland) blade with a cutting force of 80 gf, a cutting speed of 20 cm/s, off-set 0.25 and heavy as cutting quality.

For circular bonding samples of Cyclic olefin copolymer (COC) samples (COC 8007 from TekniPlex) with a height of 128 µm was punched with an 8 mm biopsy puncher to generate samples with a surface of 0.5 cm^2^.

For the assessment of the bonding strength to COC, Cyclo-Olefin-Copolymer (COP) and Polymethyl methacrylate (PMMA) sample carrier of in the size of object slides were taken (76 × 26 × 1 mm). For the bonding strength of PSA to COP and PMMA manually cut piece were taken.

Ostemere 322 crystal clear (Mercene Labs AB, Sweden) a Two-component thiol-ene-epoxy was used. The two components were weighed into a glass container under a fume hood according to the ratio specified by the manufacturer. For homogenization of the viscous components the container was vortexed for approximately 3 min. Samples were created with a 500 µm high PDMS foil as described before. Following UV exposure at a dose of 700 mJ cm^−2^, the Ostemere 322 crystal clear sample was gently delaminated from the master mold manually.

For measuring the bonding strength of membranes with PSA and OSTEMER, Whatman® Nuclepore Track-Etched polycarbonate membranes PVP-free pore size 5 μm (Sigma-Aldrich) and ipCELLCULTURE track etched polyester membrane (pore size 3 μm, pore density 8·10^5^/cm^2^, thickness 9 μm) were used.

### Bonding procedures

For determining the tensile and shear strength of each PSA sample a circular area of 0.5 cm^2^ was bonded between two glass slides with 125 N applied pressure for 1 min in a shop press WP 20 H (Holzmann Maschinen, Austria) equipped with a precision tension and compression load cell 8524 (Burster, Germany) and Digital Indicator 9163 (Burster, Germany) for force read out. For testing the strength of bonding to membranes (PET and PC) as well as to COC, COP, PMMA and PDMS the same procedure was conducted by placing a sample between two 0.5 cm^2^ PSA layers laminated on glass slides.

PDMS was bonded either to PDMS or to glass by the following procedure: the surfaces which are needed to be bond together were activated with air plasma by an Expanded Plasma Cleaner PDC-001 from Harrick Plasma for 2 min with the power setting “high” and cured for 10 min at 80 °C with a pressure of 60 N/cm^2^. To assess the bonding strength of PDMS to PDMS square shaped patches of 26 × 26 mm were first bonded to glass and then a circular sample of PDMS was bonded in between. Therefore, the bonding surface of PDMS to glass can be neglected because it is 13.5 time bigger compared to the crucial surface of the circular sample.

For solvent bonding of COC to COC and COP, first Cyclohexane was diluted with Acetone 3:1, then COC samples were placed on top of a soaked filter membrane from VWR, Austria for 1 min. Afterwards the sample were washed with isopropanol. After the placement of the COC sample between two sample carrier either both from COC or COP and rested at room temperature with a pressure of 60 N/cm^2^ for 5 min.

For plasma bonding combined with silanizaton for bonding first PDMS sheets of 26mm × 26 mm where bonded to glass as described before and PET membrane samples in the same size were used. 5% APTES solutions were prepared in pure Ethanol, the PET samples were plasma treated for 20 sec. with the settings as mentioned before and then the PET sample was immersed for 1 min. in the silane solution and dried at room temperature. The PDMS sheet was plasma activate for 30 sec. and then cured at 70 °C overnight.

Ostemer 322 crystal clear was either directly bonded to Ostemer 322 crystal clear, PET and PC membranes through a hard bake after curing at 90 °C for 90 min and an applied pressure of 20 N/cm^2^. For bonding to glass and PDMS, glass and PDMS were pretreated with (3-Aminopropyl) triethoxysilane (APTES) given by the following protocol: first APTES was diluted to concentration of 5% in 9:1 100% Ethanol/DI H_2_O and vortex till mixture become clear. After the air plasma activation of the PDMS and the glass as described before the solution was applied on the surface for 15 min at room temperature. Afterwards the APTES solution was removed and the samples were washed with pure Ethanol and dry blown. Finally, the samples were bonded to OSTEMER at 110 °C for 1 h with a pressure of 20 N/cm^2^. To assess the bonding strength of PDMS, PET and PC to Ostemere 322 crystal clear, square shaped patches of 26 × 26 mm were first bonded to glass and then a circular sample of each material was bonded in between. Therefore, the bonding surface of Ostemere 322 crystal clear to glass can be neglected because it is 13.5 time bigger compared to the crucial surface of the circular sample.

### Assessment of tensile and shear stress

To assess the bonding strength Force was applied with a shop press WP 20 H (Holzmann Maschinen, Austria) equipped with a precision tension and compression load cell 8524 (Burster, Germany) and Digital Indicator 9163 (Burster, Germany) for peak force read out. Force was applied by a rate of approximately 10 N/s. The peak force measured by the sensor was used to determine the force to failure for each sample. After each cycle, delamination of the samples from the material of interest was checked and samples delaminated otherwise in the set-up were excluded. To evaluate bonding after a period of incubation, samples were incubated for 7d at 37 °C and humified atmosphere.

### Investigation of tensile and shear bonding strength of advanced microfluidic and biochip structures

To investigate the bonding strength of advanced microfluidic and biochip structures two established and published systems were used. To evaluate the bonding strength of cell-based microfluidic device composed of glass and biocompatible pressure sensitive adhesive tapes (ARseal 90880 and ARcare 8259) the design and fabrication used as previousl^29^. The single channel system was manufactured with in base dimensions of 20 × 15 mm to be mounted within the device. For the assessment of a PDMS/glass based microfluidic biochips the device of Bachmann *et al*. was built with in the base dimensions of 26 × 13 mm as previously described^30^.

### Statistical analysis

Statistical analysis to evaluated significance was carried out with GraphPad Prism two-tailed student’s T-test.

## Supplementary information

Supplementary Information.

## References

[1] Bartholomeusz DA, Boutté RW, Andrade JD (2005). Xurography: rapid prototyping of microstructures using a cutting plotter. Journal of Microelectromechanical systems.

[2] Xia Y, Whitesides GM (1998). Soft lithography. Annual review of materials science.

[3] Rothbauer M, Rosser JM, Zirath H, Ertl P (2018). Tomorrow today: organ-on-a-chip advances towards clinically relevant pharmaceutical and medical *in vitro* models. Current opinion in biotechnology.

[4] Rothbauer M, Wartmann D, Charwat V, Ertl P (2015). Recent advances and future applications of microfluidic live-cell microarrays. Biotechnology advances.

[5] Rothbauer M, Zirath H, Ertl P (2018). Recent advances in microfluidic technologies for cell-to-cell interaction studies. Lab on a Chip.

[6] Ergir EE, Bachmann B, Redl HR, Forte G, Ertl P (2018). Small force, big impact: next generation organ-on-a-chip systems incorporating biomechanical cues. Frontiers in physiology.

[7] Sin A (2004). The design and fabrication of three-chamber microscale cell culture analog devices with integrated dissolved oxygen sensors. Biotechnology progress.

[8] Zhang B, Korolj A, Lai BFL, Radisic M (2018). Advances in organ-on-a-chip engineering. *Nature Reviews*. Materials.

[9] Harrison DJ, Manz A, Fan Z, Luedi H, Widmer HM (1992). Capillary electrophoresis and sample injection systems integrated on a planar glass chip. Analytical chemistry.

[10] Manz A (1992). Planar chips technology for miniaturization and integration of separation techniques into monitoring systems: capillary electrophoresis on a chip. Journal of Chromatography A.

[11] Huh D (2010). Reconstituting organ-level lung functions on a chip. Science.

[12] Lee H, Cho D-W (2016). One-step fabrication of an organ-on-a-chip with spatial heterogeneity using a 3D bioprinting technology. Lab on a Chip.

[13] Sticker D, Rothbauer M, Lechner S, Hehenberger M-T, Ertl P (2015). Multi-layered, membrane-integrated microfluidics based on replica molding of a thiol–ene epoxy thermoset for organ-on-a-chip applications. Lab on a Chip.

[14] Novak R, Ranu N, Mathies RA (2013). Rapid fabrication of nickel molds for prototyping embossed plastic microfluidic devices. Lab on a Chip.

[15] Pemg BY, Wu CW, Shen YK, Lin Y (2010). Microfluidic chip fabrication using hot embossing and thermal bonding of COP. Polymers for Advanced Technologies.

[16] Ahadian S (2018). Organ-on-a-chip platforms: a convergence of advanced materials, cells, and microscale technologies. Advanced healthcare materials.

[17] Ren K, Zhou J, Wu H (2013). Materials for microfluidic chip fabrication. Accounts of chemical research.

[18] Uto K, Tsui JH, DeForest CA, Kim D-H (2017). Dynamically tunable cell culture platforms for tissue engineering and mechanobiology. Progress in polymer science.

[19] Halldorsson S, Lucumi E, Gómez-Sjöberg R, Fleming RM (2015). Advantages and challenges of microfluidic cell culture in polydimethylsiloxane devices. Biosensors and Bioelectronics.

[20] Gu P, Liu K, Chen H, Nishida T, Fan ZH (2010). Chemical-assisted bonding of thermoplastics/elastomer for fabricating microfluidic valves. Analytical chemistry.

[21] Tang L, Lee NY (2010). A facile route for irreversible bonding of plastic-PDMS hybrid microdevices at room temperature. Lab on a Chip.

[22] Sip CG, Folch A (2014). Stable chemical bonding of porous membranes and poly (dimethylsiloxane) devices for long-term cell culture. Biomicrofluidics.

[23] Jerabek M, Major Z, Lang RW (2010). Uniaxial compression testing of polymeric materials. Polymer testing.

[24] Zhang H, Lee NY (2015). Non-silicon substrate bonding mediated by poly (dimethylsiloxane) interfacial coating. Applied Surface Science.

[25] Kratz S (2019). Characterization of four functional biocompatible pressure-sensitive adhesives for rapid prototyping of cell-based lab-on-a-chip and organ-on-a-chip systems. Scientific Reports.

[26] Bhattacharya S, Datta A, Berg JM, Gangopadhyay S (2005). Studies on surface wettability of poly (dimethyl) siloxane (PDMS) and glass under oxygen-plasma treatment and correlation with bond strength. Journal of microelectromechanical systems.

[27] Keller N (2016). Tacky cyclic olefin copolymer: a biocompatible bonding technique for the fabrication of microfluidic channels in COC. Lab on a Chip.

[28] Sticker D (2019). Oxygen Management at the Microscale: A Functional Biochip Material with Long-Lasting and Tunable Oxygen Scavenging Properties for Cell Culture Applications. ACS applied materials & interfaces.

[29] Zirath H (2018). Every breath you take: non-invasive real-time oxygen biosensing in two-and three-dimensional microfluidic cell models. Frontiers in physiology.

[30] Bachmann B (2018). Engineering of three-dimensional pre-vascular networks within fibrin hydrogel constructs by microfluidic control over reciprocal cell signaling. Biomicrofluidics.

[31] Jia Z-J, Fang Q, Fang Z-L (2004). Bonding of glass microfluidic chips at room temperatures. Analytical chemistry.

[32] Waldbaur A, Rapp H, Länge K, Rapp BE (2011). Let there be chip—towards rapid prototyping of microfluidic devices: one-step manufacturing processes. Analytical Methods.

[33] Zhou XC (2017). Thiol–ene–epoxy thermoset for low-temperature bonding to biofunctionalized microarray surfaces. Lab on a Chip.

[34] Chang Z, Yang R, Wei Y (2019). The linear-dependence of adhesion strength and adhesion range on temperature in soft membranes. Journal of the Mechanics and Physics of Solids.

[35] Tsao C-W, DeVoe DL (2009). Bonding of thermoplastic polymer microfluidics. Microfluidics and nanofluidics.

[36] Wei Y (2014). A stochastic description on the traction-separation law of an interface with non-covalent bonding. Journal of the Mechanics and Physics of Solids.

[37] Volpatti LR, Yetisen AK (2014). Commercialization of microfluidic devices. Trends in biotechnology.

[38] Sollier E, Murray C, Maoddi P, Di Carlo D (2011). Rapid prototyping polymers for microfluidic devices and high pressure injections. Lab on a Chip.

[39] Berthier E, Young EW, Beebe D (2012). Engineers are from PDMS-land, Biologists are from Polystyrenia. Lab on a Chip.

