# Supplementary material for: A compression transmission device for the evaluation of bonding strength of biocompatible microfluidic and biochip materials and systems
Source: Sci Rep. 2020 Jan 29;10:1400. doi: 10.1038/s41598-020-58373-0 (PMC6989640; doi:10.1038/s41598-020-58373-0)
Supplement: Supplementary file 1 — Supplementary Information. [file 41598_2020_58373_MOESM1_ESM.docx]

**Supporting information - A compression transmission device for the evaluation of bonding strength of biocompatible microfluidic and biochip materials and systems**

S.R.A. Kratz ^1,2^, B. Bachmann ^1,2,3^, S. Spitz ^1,2^, Gregor Höll^1,2^, C. Eilenberger ^1,2^ Hannah Goeritz^4^, P. Ertl ^1,2^ and M. Rothbauer^1,2^

*^1^ Institute of Applied Synthetic Chemistry and Institute of Chemical Technologies and Analytics, Faculty of Technical Chemistry, Vienna University of Technology, Getreidemarkt 9/163-164, 1060 Vienna, Austria*

*^2^ Austrian Cluster for Tissue Regeneration, Vienna, Austria*

*^3^ Ludwig Boltzmann Institute for Experimental and Clinical Traumatology, Allgemeine Unfallversicherungsanstalt (AUVA) Research Centre, Donaueschingenstraße 13, 1200 Vienna, Austria*

*^4^ Institute of Solid State Electronics, Faculty of Electrical Engineering and Information Technology, Vienna University of Technology, Gußhausstraße 25-25a, 1040 Vienna, Austria*

**
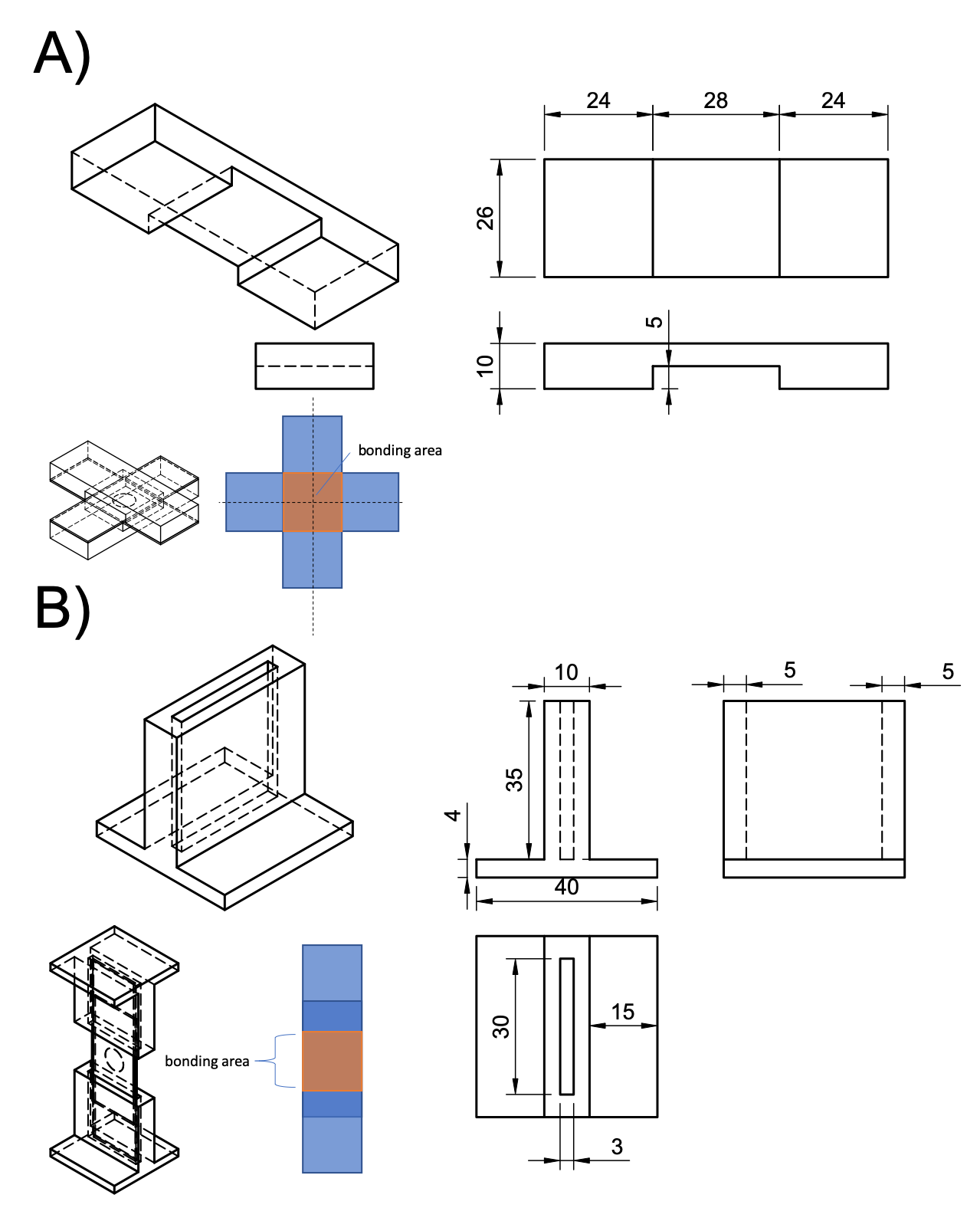
**

**SI-Figure** **1** engineering drawing of device to test method for evaluation of A) tensile and B) shear bonding strength with device including mounted sample. All dimensions are given in mm.


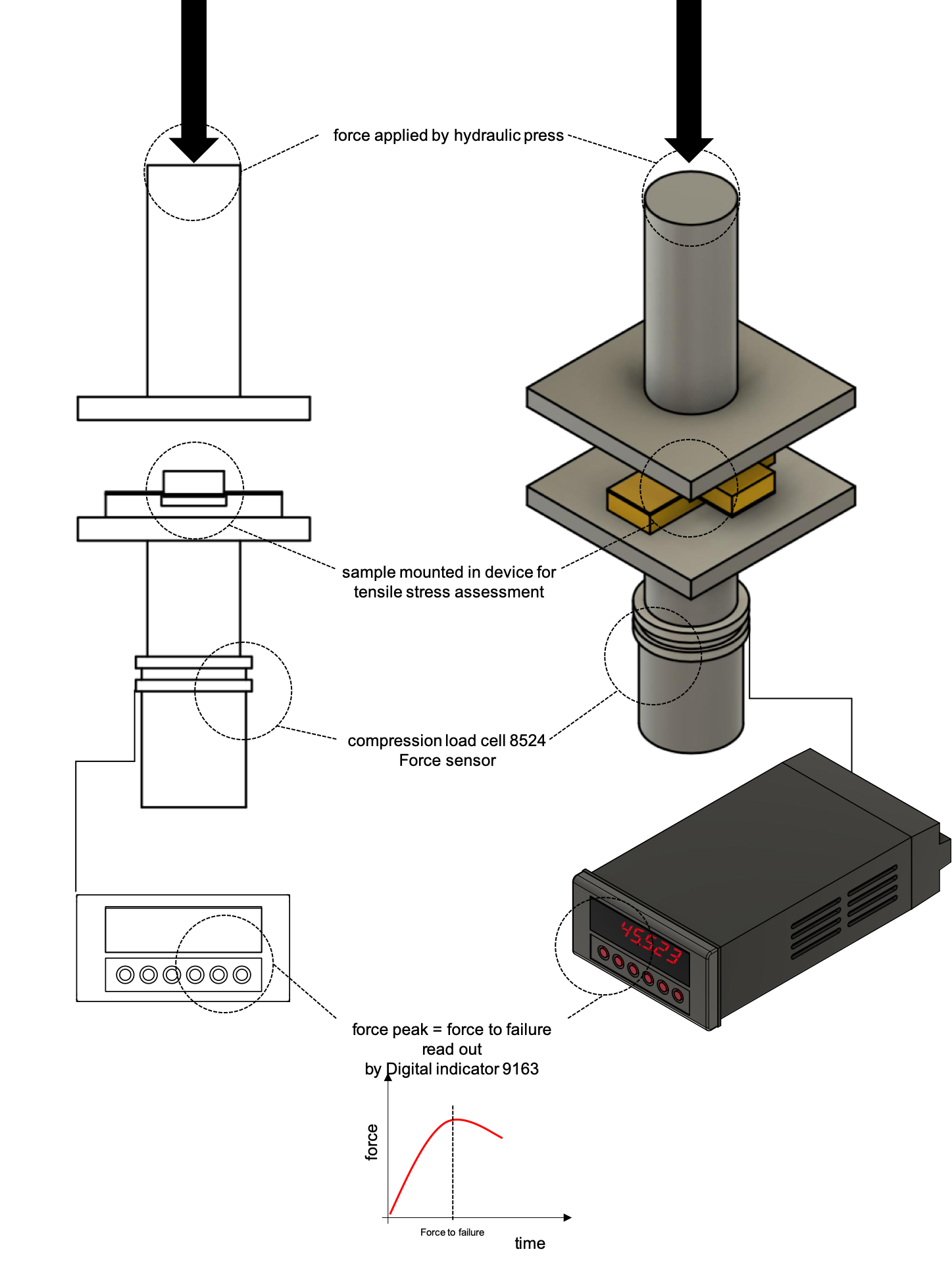


**SI-Figure** **2** Set-up for compression test method for evaluation of tensile and shear bonding strength with device including mounted sample within a hydraulic press equipped with a compression force sensor and peak force read out
